# Ethyl 1-(4-methoxy­phen­yl)-2-nitro-3-[4-oxo-3-phenyl-1-(4-methoxy­phen­yl)azetidin-2-yl]-2,3,10,10a-tetra­hydro-1*H*,5*H*-pyrrolo[1,2-*b*]isoquinoline-10a-carboxyl­ate

**DOI:** 10.1107/S1600536808010428

**Published:** 2008-04-23

**Authors:** E. Theboral Sugi Kamala, S. Nirmala, L. Sudha, N. Arumugam, R. Raghunathan

**Affiliations:** a Department of Physics, Easwari Engineering College, Ramapuram, Chennai 600 089, India; b Department of Physics, SRM University, Ramapuram Campus, Chennai 600 089, India; c Department of Organic Chemistry, University of Madras, Guindy Campus, Chennai 600 025, India

## Abstract

In the mol­ecule of the title compound, C_38_H_37_N_3_O_7_, the pyrrolidine ring adopts a twist conformation and the six-membered heterocyclic ring has a boat conformation. In the crystal structure, mol­ecules are linked into a three-dimensional framework through inter­molecular C—H⋯O hydrogen bonds. One ethyl group is disordered over two positions with occupancies 0.67 (2)/0.33 (2).

## Related literature

For related literature, see: Allen *et al.* (1987 ▶); Amal Raj *et al.* (2003 ▶); Borthwick *et al.* (2003 ▶); Brakhage (1998 ▶); Cremer & Pople (1975 ▶); Fernandes *et al.* (2004 ▶); Kamala *et al.* (2008 ▶); Katritzky *et al.* (1996 ▶); Morin & Gorman (1982 ▶); Nardelli (1983 ▶); Sundari *et al.* (2006 ▶); Verkman (1990 ▶); Weissman *et al.* (1993 ▶); Georg & Ravikumar (1993 ▶); LaVoie *et al.* (1983 ▶).
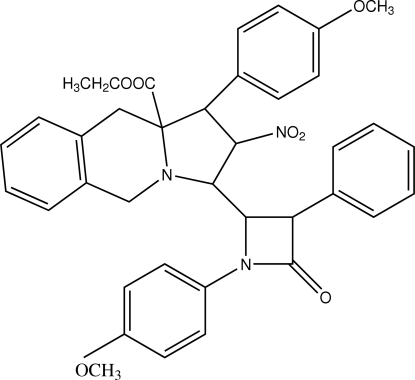


## Experimental

### 

#### Crystal data

C_38_H_37_N_3_O_7_
*M* *_r_* = 647.71
Orthorhombic, 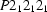

*a* = 9.0149 (3) Å
*b* = 11.0865 (4) Å
*c* = 33.3731 (11) Å
*V* = 3335.4 (2) Å^3^
*Z* = 4
Mo *K*α radiation
μ = 0.09 mm^−1^
*T* = 293 (2) K
0.20 × 0.20 × 0.20 mm

#### Data collection

Bruker Kappa APEX2 diffractometer
Absorption correction: multi-scan (Blessing, 1995 ▶) *T*_min_ = 0.982, *T*_max_ = 0.982
68599 measured reflections
3960 independent reflections
3318 reflections with *I* > 2σ(*I*)
*R*_int_ = 0.033

#### Refinement

*R*[*F* ^2^ > 2σ(*F* ^2^)] = 0.034
*wR*(*F* ^2^) = 0.107
*S* = 1.05
3960 reflections
453 parameters
H-atom parameters constrained
Δρ_max_ = 0.14 e Å^−3^
Δρ_min_ = −0.16 e Å^−3^

### 

Data collection: *APEX2* (Bruker, 2004 ▶); cell refinement: *APEX2* and *SAINT* (Bruker, 2004 ▶); data reduction: *SAINT* and *XPREP* Bruker, 2004 ▶); program(s) used to solve structure: *SHELXS97* (Sheldrick, 2008 ▶); program(s) used to refine structure: *SHELXL97* (Sheldrick, 2008 ▶); molecular graphics: *ORTEP-3* (Farrugia, 1997 ▶); software used to prepare material for publication: *PLATON* (Spek, 2003 ▶).

## Supplementary Material

Crystal structure: contains datablocks I, global. DOI: 10.1107/S1600536808010428/bt2696sup1.cif

Structure factors: contains datablocks I. DOI: 10.1107/S1600536808010428/bt2696Isup2.hkl

Additional supplementary materials:  crystallographic information; 3D view; checkCIF report

## Figures and Tables

**Table 1:** Hydrogen-bond geometry (Å, °)

*D*—H⋯*A*	*D*—H	H⋯*A*	*D*⋯*A*	*D*—H⋯*A*
C6—H6⋯O2^i^	0.98	2.42	3.260 (3)	143
C20—H20⋯O1^ii^	0.93	2.47	3.397 (3)	172
C29—H29⋯O3	0.93	2.59	3.442 (3)	152
C29—H29⋯N2	0.93	2.50	3.168 (3)	128
C33—H33⋯O1	0.93	2.44	3.054 (4)	123

Symmetry codes: (i) 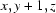; (ii) 
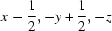
.

**Fig. 1.**
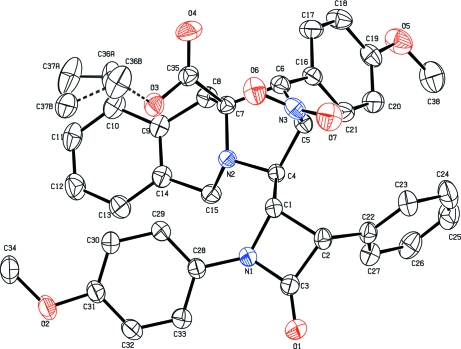
The molecular structure of (I) with 30% probability displacement ellipsoids.

**Fig. 2.**
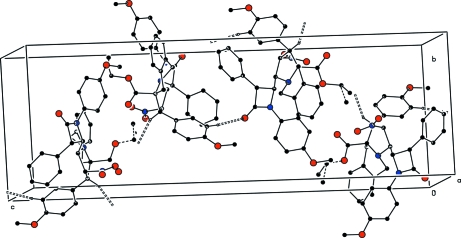
The packing of the molecules viewed along the c axis.

## supplementary crystallographic information

### Comment

Quinoline is a hepatocarcinogen in mice and rats, a mutagen in Salmonella typhimurium and induces unscheduled DNA synthesis in primary cultures of rat hepatocytes (LaVoie *et al.*, 1983). Quinolinium based chloride sensitive flourescent indicators provide a new approach to study chloride transport mechanisms and regulation (Verkman, 1990). Pyrrole derivatives inhibit cytokine-dependent induction of human immunodeficiency virus (HIV) expression in chronically infected promonocytic cells (Weissman *et al.*, 1993). Pyrroles possess anti inflammatory (Fernandes *et al.*, 2004), anti viral (Borthwick *et al.*, 2003), antifungal and antimicrobial activities (Amal Raj *et al.*, 2003). β lactams are one of the best known and most extensively studied class of compounds due to their biological activity (Morin & Gorman, 1982; Georg & Ravikumar, 1993; Katritzky *et al.*, 1996). The most commonly used β lactam antibiotics for the therapy of infectious diseases are penicillin and cephalosporin (Brakhage, 1998). Due to these importance the crystal structure determination of the title compound (I) was carried out and the results are presented here.

Fig 1 shows a plot of compound (I). Bond lengths and angles are within normal ranges (Allen *et al.*, 1987) and comparable with those of reported structures (Kamala *et al.*, 2008; Sundari *et al.*, 2006).

In the molecule the pyrrolidine ring exhibits a *twist* conformation with asymetry parameters (Nardelli,1983)ΔC_s_(C5) = 5.2 (2), ΔC_2_(C6) = 39.6 (2) and with the puckering parameters (Cremer and Pople, 1975) q2 = 0.274 (3)Å and φ2 =267.5 (5)°. The six membered ring C7/C8/C9/C14/C15/N2 exhibits a *boat* conformation with the puckering parameters Q=0.699 (3) Å, Θ = 88.8 (2)° and φ = 120.1 (2)°. The sum of bond angles around N1 [353.59°], and around N3 [359.94°] indicate *sp*^2^, those around atom N2 [338.58°] indicate *sp*^3^   hybridization. The pyrrolidine ring and the phenyl ring C9—C14 are nearly planar with each other with a dihedral angle of 9.49 (7)° while the phenyl rings are oriented at right angles to each other making an angle of 87.48 (7)°.

In the crystal packing, C—H···O interactions stabilize crystal structure.

### Experimental

To a stirring solution of 1 mmol of 5-[1'-*N*-(*p*-methoxy)-phenyl)-3'-phenyl- azetidine-2'-one]-4-nitro-3-(*p*-methoxy)-phenyl-2-ethoxy carbonyl-2-benzyl -pyrrolidine in 20 ml of dry chloroform was added 1 mmol of paraformaldehyde followed by 0.1 mmol of trifluroacetic acid at room temperature. After completion of the reaction the mixture was washed with water and dried over Na_2_SO_4_. The solvent was removed under reduced pressure and the crude product was subjected to column chromatography with hexane ethylacetate (9:1) to obtain pure cyclized product. The compound was recrystallized from ethylacetae.

### Refinement

In the absence of anomalous scatterers, Friedel pairs were merged and the absolute configuration was arbitrarily set. One ethyl group is disordered over two positions (C36A/C37A and C36B/C37B), with refined occupancies of 0.67 (2) and 0.33 (2). H atoms were placed in idealized positions and allowed to ride on their parent atoms, with C–H = 0.93 or 0.96 Å and *U*_iso_(H)= 1.2–1.5*U*_eq_(C).

### Crystal data

**Table:**

C_38_H_37_N_3_O_7_	*F*_000_ = 1368
*M**_r_* = 647.71	*D*_x_ = 1.290 Mg m^−^^3^
Orthorhombic, *P*2_1_2_1_2_1_	Mo *K*α radiation λ = 0.71073 Å
Hall symbol: P 2ac 2ab	Cell parameters from 68599 reflections
*a* = 9.0149 (3) Å	θ = 1.2–26.7º
*b* = 11.0865 (4) Å	µ = 0.09 mm^−^^1^
*c* = 33.3731 (11) Å	*T* = 293 (2) K
*V* = 3335.4 (2) Å^3^	Prism, colourless
*Z* = 4	0.20 × 0.20 × 0.20 mm

### Data collection

**Table:**

Bruker KappaAPEX2 diffractometer	3960 independent reflections
Radiation source: fine-focus sealed tube	3318 reflections with *I* > 2σ(*I*)
Monochromator: graphite	*R*_int_ = 0.033
*T* = 293(2) K	θ_max_ = 26.7º
ω and φ scans	θ_min_ = 1.2º
Absorption correction: multi-scan(Blessing, 1995)	*h* = −11→11
*T*_min_ = 0.982, *T*_max_ = 0.982	*k* = −13→13
68599 measured reflections	*l* = −42→42

### Refinement

**Table:**

Refinement on *F*^2^	Hydrogen site location: inferred from neighbouring sites
Least-squares matrix: full	H-atom parameters constrained
*R*[*F*^2^ > 2σ(*F*^2^)] = 0.034	*w* = 1/[σ^2^(*F*_o_^2^) + (0.0606*P*)^2^ + 0.4467*P*] where *P* = (*F*_o_^2^ + 2*F*_c_^2^)/3
*wR*(*F*^2^) = 0.107	(Δ/σ)_max_ < 0.001
*S* = 1.06	Δρ_max_ = 0.14 e Å^−^^3^
3960 reflections	Δρ_min_ = −0.16 e Å^−^^3^
453 parameters	Extinction correction: SHELXL97 (Sheldrick, 2008), Fc^*^=kFc[1+0.001xFc^2^λ^3^/sin(2θ)]^-1/4^
Primary atom site location: structure-invariant direct methods	Extinction coefficient: 0.0035 (7)
Secondary atom site location: difference Fourier map	

### Special details

**Table:**

Geometry. All e.s.d.'s (except the e.s.d. in the dihedral angle between two l.s. planes) are estimated using the full covariance matrix. The cell e.s.d.'s are taken into account individually in the estimation of e.s.d.'s in distances, angles and torsion angles; correlations between e.s.d.'s in cell parameters are only used when they are defined by crystal symmetry. An approximate (isotropic) treatment of cell e.s.d.'s is used for estimating e.s.d.'s involving l.s. planes.
Refinement. Refinement of *F*^2^ against ALL reflections. The weighted *R*-factor *wR* and goodness of fit *S* are based on *F*^2^, conventional *R*-factors *R* are based on *F*, with *F* set to zero for negative *F*^2^. The threshold expression of *F*^2^ > σ(*F*^2^) is used only for calculating *R*-factors(gt) *etc*. and is not relevant to the choice of reflections for refinement. *R*-factors based on *F*^2^ are statistically about twice as large as those based on *F*, and *R*- factors based on ALL data will be even larger.

### Fractional atomic coordinates and isotropic or equivalent isotropic displacement parameters (Å^2^)

**Table:**

	*x*	*y*	*z*	*U*_iso_*/*U*_eq_	Occ. (<1)
C1	0.5650 (3)	0.1895 (2)	0.11810 (7)	0.0428 (5)	
H1	0.6078	0.1997	0.1449	0.051*	
C2	0.6846 (3)	0.2110 (2)	0.08439 (7)	0.0465 (5)	
H2	0.7811	0.2300	0.0964	0.056*	
C3	0.6714 (3)	0.0759 (2)	0.07673 (7)	0.0518 (6)	
C4	0.4217 (3)	0.26005 (18)	0.11375 (6)	0.0392 (5)	
H4	0.3956	0.2613	0.0853	0.047*	
C5	0.4352 (3)	0.3919 (2)	0.12799 (7)	0.0441 (5)	
H5	0.4661	0.4435	0.1057	0.053*	
C6	0.2805 (3)	0.4279 (2)	0.14202 (7)	0.0452 (5)	
H6	0.2911	0.4792	0.1658	0.054*	
C7	0.2091 (3)	0.3061 (2)	0.15540 (6)	0.0418 (5)	
C8	0.0419 (3)	0.2946 (2)	0.14583 (8)	0.0509 (6)	
H8A	−0.0144	0.3490	0.1628	0.061*	
H8B	0.0240	0.3164	0.1181	0.061*	
C9	−0.0065 (3)	0.1674 (2)	0.15306 (7)	0.0481 (6)	
C10	−0.1225 (3)	0.1330 (3)	0.17775 (8)	0.0601 (7)	
H10	−0.1790	0.1911	0.1908	0.072*	
C11	−0.1538 (4)	0.0124 (3)	0.18292 (9)	0.0702 (8)	
H11	−0.2323	−0.0106	0.1993	0.084*	
C12	−0.0702 (4)	−0.0739 (3)	0.16414 (10)	0.0740 (9)	
H12	−0.0926	−0.1551	0.1677	0.089*	
C13	0.0469 (4)	−0.0409 (3)	0.13991 (9)	0.0645 (7)	
H13	0.1039	−0.0996	0.1273	0.077*	
C14	0.0792 (3)	0.0797 (2)	0.13448 (7)	0.0505 (6)	
C15	0.2094 (3)	0.1248 (2)	0.11103 (7)	0.0480 (5)	
H15A	0.2717	0.0576	0.1032	0.058*	
H15B	0.1752	0.1650	0.0869	0.058*	
C16	0.1960 (3)	0.4982 (2)	0.11054 (7)	0.0469 (5)	
C17	0.0909 (3)	0.5824 (2)	0.12263 (8)	0.0601 (7)	
H17	0.0744	0.5938	0.1499	0.072*	
C18	0.0110 (4)	0.6489 (2)	0.09578 (9)	0.0658 (7)	
H18	−0.0600	0.7033	0.1048	0.079*	
C19	0.0358 (3)	0.6354 (2)	0.05499 (8)	0.0592 (7)	
C20	0.1387 (3)	0.5535 (3)	0.04209 (8)	0.0598 (7)	
H20	0.1563	0.5437	0.0148	0.072*	
C21	0.2168 (3)	0.4850 (2)	0.06968 (7)	0.0550 (6)	
H21	0.2852	0.4286	0.0605	0.066*	
C22	0.6510 (3)	0.2965 (2)	0.05059 (7)	0.0484 (5)	
C23	0.7102 (4)	0.4110 (3)	0.05080 (9)	0.0628 (7)	
H23	0.7770	0.4333	0.0707	0.075*	
C24	0.6704 (4)	0.4931 (3)	0.02139 (11)	0.0822 (10)	
H24	0.7105	0.5704	0.0218	0.099*	
C25	0.5729 (4)	0.4619 (3)	−0.00828 (10)	0.0822 (10)	
H25	0.5448	0.5181	−0.0275	0.099*	
C26	0.5175 (4)	0.3476 (3)	−0.00931 (8)	0.0732 (9)	
H26	0.4531	0.3252	−0.0298	0.088*	
C27	0.5563 (3)	0.2648 (3)	0.01987 (7)	0.0563 (6)	
H27	0.5182	0.1869	0.0188	0.068*	
C28	0.5268 (3)	−0.0417 (2)	0.12839 (7)	0.0471 (5)	
C29	0.4518 (3)	−0.0313 (2)	0.16414 (8)	0.0549 (6)	
H29	0.4239	0.0446	0.1733	0.066*	
C30	0.4176 (3)	−0.1326 (2)	0.18659 (8)	0.0579 (7)	
H30	0.3682	−0.1244	0.2109	0.069*	
C31	0.4563 (4)	−0.2445 (2)	0.17306 (8)	0.0583 (7)	
C32	0.5345 (4)	−0.2552 (2)	0.13739 (8)	0.0698 (8)	
H32	0.5635	−0.3311	0.1286	0.084*	
C33	0.5695 (4)	−0.1558 (2)	0.11504 (8)	0.0615 (7)	
H33	0.6214	−0.1642	0.0911	0.074*	
C34	0.3451 (6)	−0.3423 (3)	0.22891 (10)	0.1026 (15)	
H34A	0.3317	−0.4215	0.2399	0.154*	
H34B	0.2500	−0.3067	0.2236	0.154*	
H34C	0.3984	−0.2930	0.2477	0.154*	
C35	0.2155 (3)	0.2959 (2)	0.20132 (7)	0.0509 (6)	
C36A	0.227 (2)	0.1693 (8)	0.2565 (2)	0.099 (4)	0.67 (2)
H36A	0.3178	0.1371	0.2677	0.119*	0.67 (2)
H36B	0.2132	0.2487	0.2681	0.119*	0.67 (2)
C36B	0.2763 (18)	0.166 (2)	0.2577 (7)	0.094 (8)	0.33 (2)
H36C	0.2370	0.2319	0.2737	0.113*	0.33 (2)
H36D	0.3801	0.1541	0.2641	0.113*	0.33 (2)
C37A	0.1243 (12)	0.1053 (17)	0.2693 (3)	0.113 (5)	0.67 (2)
H37A	0.1328	0.0977	0.2978	0.170*	0.67 (2)
H37B	0.1300	0.0269	0.2572	0.170*	0.67 (2)
H37C	0.0309	0.1419	0.2627	0.170*	0.67 (2)
C37B	0.182 (4)	0.0452 (14)	0.2633 (4)	0.130 (9)	0.33 (2)
H37D	0.1729	0.0272	0.2914	0.196*	0.33 (2)
H37E	0.2311	−0.0203	0.2500	0.196*	0.33 (2)
H37F	0.0849	0.0560	0.2520	0.196*	0.33 (2)
C38	−0.0228 (5)	0.6993 (3)	−0.01129 (10)	0.0934 (11)	
H38A	−0.0871	0.7543	−0.0252	0.140*	
H38B	−0.0431	0.6184	−0.0199	0.140*	
H38C	0.0786	0.7190	−0.0171	0.140*	
N1	0.5645 (2)	0.06099 (17)	0.10584 (6)	0.0476 (5)	
N2	0.2955 (2)	0.20995 (16)	0.13575 (5)	0.0402 (4)	
N3	0.5465 (3)	0.40018 (18)	0.16171 (7)	0.0531 (5)	
O1	0.7299 (3)	0.00518 (18)	0.05473 (6)	0.0730 (6)	
O2	0.4270 (3)	−0.35051 (16)	0.19254 (5)	0.0772 (7)	
O3	0.2539 (2)	0.18744 (16)	0.21413 (5)	0.0614 (5)	
O4	0.1785 (3)	0.37724 (19)	0.22274 (6)	0.0777 (6)	
O5	−0.0475 (3)	0.70850 (19)	0.03026 (7)	0.0815 (6)	
O6	0.5102 (2)	0.3667 (2)	0.19483 (6)	0.0699 (6)	
O7	0.6700 (2)	0.4365 (2)	0.15328 (7)	0.0782 (6)	

### Atomic displacement parameters (Å^2^)

**Table:**

	*U*^11^	*U*^22^	*U*^33^	*U*^12^	*U*^13^	*U*^23^
C1	0.0449 (12)	0.0404 (11)	0.0429 (11)	−0.0039 (10)	0.0002 (10)	0.0047 (9)
C2	0.0392 (11)	0.0484 (13)	0.0519 (12)	−0.0036 (10)	0.0007 (10)	0.0082 (10)
C3	0.0535 (14)	0.0512 (13)	0.0508 (13)	0.0027 (12)	0.0079 (11)	0.0064 (11)
C4	0.0432 (11)	0.0373 (10)	0.0371 (10)	−0.0031 (9)	−0.0003 (9)	0.0023 (9)
C5	0.0466 (12)	0.0394 (11)	0.0464 (11)	−0.0039 (10)	−0.0068 (10)	0.0027 (9)
C6	0.0509 (13)	0.0396 (11)	0.0452 (12)	−0.0006 (11)	−0.0026 (10)	−0.0048 (10)
C7	0.0449 (11)	0.0428 (11)	0.0377 (10)	0.0001 (11)	−0.0014 (9)	−0.0024 (9)
C8	0.0424 (12)	0.0563 (14)	0.0540 (13)	0.0024 (12)	−0.0023 (11)	0.0022 (11)
C9	0.0394 (12)	0.0631 (15)	0.0419 (11)	−0.0043 (11)	−0.0057 (10)	0.0048 (11)
C10	0.0426 (13)	0.0831 (19)	0.0546 (14)	−0.0019 (14)	−0.0012 (12)	0.0051 (14)
C11	0.0568 (16)	0.091 (2)	0.0629 (16)	−0.0254 (17)	0.0044 (14)	0.0133 (16)
C12	0.074 (2)	0.0660 (18)	0.082 (2)	−0.0271 (18)	0.0046 (17)	0.0048 (16)
C13	0.0657 (18)	0.0564 (15)	0.0716 (17)	−0.0199 (15)	0.0026 (15)	−0.0070 (13)
C14	0.0469 (13)	0.0582 (14)	0.0463 (12)	−0.0132 (12)	−0.0050 (10)	−0.0018 (11)
C15	0.0487 (13)	0.0488 (12)	0.0464 (12)	−0.0099 (11)	0.0007 (11)	−0.0088 (10)
C16	0.0530 (14)	0.0380 (11)	0.0498 (12)	−0.0001 (11)	−0.0011 (11)	−0.0026 (10)
C17	0.0734 (18)	0.0538 (14)	0.0531 (14)	0.0148 (14)	−0.0002 (13)	−0.0067 (12)
C18	0.0713 (18)	0.0537 (15)	0.0723 (17)	0.0186 (14)	−0.0074 (15)	−0.0066 (14)
C19	0.0673 (17)	0.0438 (13)	0.0665 (15)	0.0025 (13)	−0.0126 (14)	0.0081 (12)
C20	0.0722 (17)	0.0584 (15)	0.0488 (13)	0.0083 (15)	−0.0034 (13)	0.0048 (11)
C21	0.0643 (16)	0.0488 (13)	0.0520 (13)	0.0105 (13)	0.0008 (12)	0.0006 (11)
C22	0.0410 (12)	0.0519 (13)	0.0524 (13)	−0.0002 (11)	0.0046 (10)	0.0104 (11)
C23	0.0612 (16)	0.0583 (16)	0.0689 (16)	−0.0106 (14)	−0.0019 (14)	0.0140 (13)
C24	0.093 (2)	0.0612 (18)	0.093 (2)	−0.0071 (18)	0.005 (2)	0.0275 (17)
C25	0.085 (2)	0.089 (2)	0.0730 (19)	0.013 (2)	0.0069 (18)	0.0370 (18)
C26	0.0676 (18)	0.102 (2)	0.0499 (14)	0.0056 (18)	−0.0012 (13)	0.0150 (16)
C27	0.0539 (15)	0.0645 (15)	0.0506 (13)	−0.0039 (13)	0.0025 (12)	0.0048 (12)
C28	0.0497 (13)	0.0423 (12)	0.0493 (12)	−0.0023 (11)	0.0018 (11)	0.0062 (10)
C29	0.0641 (16)	0.0421 (12)	0.0584 (14)	0.0028 (12)	0.0105 (13)	0.0037 (11)
C30	0.0681 (17)	0.0518 (14)	0.0537 (14)	−0.0077 (14)	0.0097 (13)	0.0072 (11)
C31	0.0806 (19)	0.0440 (13)	0.0504 (13)	−0.0154 (14)	−0.0013 (14)	0.0043 (11)
C32	0.110 (3)	0.0406 (13)	0.0590 (15)	−0.0050 (16)	0.0063 (17)	−0.0015 (12)
C33	0.085 (2)	0.0484 (14)	0.0507 (13)	−0.0015 (14)	0.0103 (14)	0.0000 (11)
C34	0.168 (4)	0.074 (2)	0.0659 (19)	−0.049 (3)	0.029 (2)	0.0030 (17)
C35	0.0561 (14)	0.0558 (14)	0.0406 (11)	−0.0051 (13)	0.0009 (11)	−0.0045 (11)
C36A	0.185 (11)	0.086 (5)	0.027 (3)	−0.057 (6)	−0.007 (5)	0.006 (3)
C36B	0.052 (6)	0.17 (2)	0.066 (9)	0.037 (11)	−0.012 (5)	0.039 (9)
C37A	0.100 (6)	0.179 (13)	0.062 (4)	−0.039 (7)	0.002 (4)	0.032 (6)
C37B	0.26 (3)	0.073 (9)	0.056 (6)	0.017 (12)	0.060 (12)	0.022 (6)
C38	0.108 (3)	0.091 (2)	0.082 (2)	0.013 (2)	−0.021 (2)	0.0345 (18)
N1	0.0521 (11)	0.0395 (10)	0.0512 (10)	0.0007 (9)	0.0073 (9)	0.0045 (8)
N2	0.0409 (10)	0.0394 (9)	0.0404 (9)	−0.0045 (8)	0.0009 (8)	−0.0033 (8)
N3	0.0540 (13)	0.0427 (10)	0.0625 (13)	−0.0060 (10)	−0.0127 (11)	−0.0022 (10)
O1	0.0886 (15)	0.0584 (11)	0.0719 (12)	0.0103 (11)	0.0320 (12)	0.0013 (10)
O2	0.1283 (19)	0.0457 (10)	0.0576 (10)	−0.0274 (12)	0.0074 (12)	0.0055 (8)
O3	0.0868 (13)	0.0590 (10)	0.0386 (8)	−0.0079 (10)	−0.0050 (9)	0.0070 (8)
O4	0.1063 (18)	0.0765 (13)	0.0502 (10)	0.0096 (13)	0.0095 (11)	−0.0168 (10)
O5	0.0965 (16)	0.0656 (12)	0.0824 (14)	0.0216 (13)	−0.0214 (13)	0.0100 (11)
O6	0.0764 (13)	0.0744 (13)	0.0590 (11)	−0.0144 (11)	−0.0207 (10)	0.0107 (10)
O7	0.0528 (12)	0.0899 (15)	0.0921 (14)	−0.0189 (12)	−0.0117 (11)	−0.0037 (12)

### Geometric parameters (Å, °)

**Table:**

C1—N1	1.482 (3)	C22—C23	1.377 (4)
C1—C4	1.517 (3)	C22—C27	1.380 (4)
C1—C2	1.577 (3)	C23—C24	1.386 (4)
C1—H1	0.9800	C23—H23	0.9300
C2—C22	1.504 (3)	C24—C25	1.369 (5)
C2—C3	1.524 (4)	C24—H24	0.9300
C2—H2	0.9800	C25—C26	1.362 (5)
C3—O1	1.197 (3)	C25—H25	0.9300
C3—N1	1.378 (3)	C26—C27	1.384 (4)
C4—N2	1.464 (3)	C26—H26	0.9300
C4—C5	1.542 (3)	C27—H27	0.9300
C4—H4	0.9800	C28—C29	1.376 (4)
C5—N3	1.511 (3)	C28—C33	1.395 (3)
C5—C6	1.524 (3)	C28—N1	1.406 (3)
C5—H5	0.9800	C29—C30	1.384 (3)
C6—C16	1.514 (3)	C29—H29	0.9300
C6—C7	1.561 (3)	C30—C31	1.366 (4)
C6—H6	0.9800	C30—H30	0.9300
C7—N2	1.474 (3)	C31—O2	1.369 (3)
C7—C35	1.538 (3)	C31—C32	1.388 (4)
C7—C8	1.545 (3)	C32—C33	1.367 (4)
C8—C9	1.496 (4)	C32—H32	0.9300
C8—H8A	0.9700	C33—H33	0.9300
C8—H8B	0.9700	C34—O2	1.424 (4)
C9—C10	1.385 (4)	C34—H34A	0.9600
C9—C14	1.388 (4)	C34—H34B	0.9600
C10—C11	1.377 (4)	C34—H34C	0.9600
C10—H10	0.9300	C35—O4	1.198 (3)
C11—C12	1.370 (5)	C35—O3	1.322 (3)
C11—H11	0.9300	C36A—C37A	1.240 (17)
C12—C13	1.380 (4)	C36A—O3	1.448 (8)
C12—H12	0.9300	C36A—H36A	0.9700
C13—C14	1.380 (4)	C36A—H36B	0.9700
C13—H13	0.9300	C36B—O3	1.49 (2)
C14—C15	1.497 (3)	C36B—C37B	1.60 (3)
C15—N2	1.474 (3)	C36B—H36C	0.9700
C15—H15A	0.9700	C36B—H36D	0.9700
C15—H15B	0.9700	C37A—H37A	0.9600
C16—C21	1.384 (3)	C37A—H37B	0.9600
C16—C17	1.390 (3)	C37A—H37C	0.9600
C17—C18	1.366 (4)	C37B—H37D	0.9600
C17—H17	0.9300	C37B—H37E	0.9600
C18—C19	1.387 (4)	C37B—H37F	0.9600
C18—H18	0.9300	C38—O5	1.408 (4)
C19—C20	1.368 (4)	C38—H38A	0.9600
C19—O5	1.379 (3)	C38—H38B	0.9600
C20—C21	1.385 (4)	C38—H38C	0.9600
C20—H20	0.9300	N3—O6	1.211 (3)
C21—H21	0.9300	N3—O7	1.217 (3)
			
N1—C1—C4	117.82 (19)	C23—C22—C2	119.9 (2)
N1—C1—C2	87.16 (17)	C27—C22—C2	121.4 (2)
C4—C1—C2	115.87 (17)	C22—C23—C24	120.1 (3)
N1—C1—H1	111.3	C22—C23—H23	120.0
C4—C1—H1	111.3	C24—C23—H23	120.0
C2—C1—H1	111.3	C25—C24—C23	120.8 (3)
C22—C2—C3	118.6 (2)	C25—C24—H24	119.6
C22—C2—C1	119.5 (2)	C23—C24—H24	119.6
C3—C2—C1	85.25 (17)	C26—C25—C24	119.3 (3)
C22—C2—H2	110.4	C26—C25—H25	120.4
C3—C2—H2	110.4	C24—C25—H25	120.4
C1—C2—H2	110.4	C25—C26—C27	120.4 (3)
O1—C3—N1	131.5 (2)	C25—C26—H26	119.8
O1—C3—C2	135.4 (2)	C27—C26—H26	119.8
N1—C3—C2	93.13 (19)	C22—C27—C26	120.7 (3)
N2—C4—C1	114.74 (16)	C22—C27—H27	119.7
N2—C4—C5	105.44 (17)	C26—C27—H27	119.7
C1—C4—C5	113.11 (18)	C29—C28—C33	119.2 (2)
N2—C4—H4	107.7	C29—C28—N1	121.0 (2)
C1—C4—H4	107.7	C33—C28—N1	119.8 (2)
C5—C4—H4	107.7	C28—C29—C30	120.7 (2)
N3—C5—C6	111.29 (19)	C28—C29—H29	119.6
N3—C5—C4	109.85 (18)	C30—C29—H29	119.6
C6—C5—C4	105.74 (18)	C31—C30—C29	120.1 (2)
N3—C5—H5	110.0	C31—C30—H30	120.0
C6—C5—H5	110.0	C29—C30—H30	120.0
C4—C5—H5	110.0	C30—C31—O2	125.0 (2)
C16—C6—C5	112.48 (19)	C30—C31—C32	119.4 (2)
C16—C6—C7	115.8 (2)	O2—C31—C32	115.6 (2)
C5—C6—C7	103.80 (18)	C33—C32—C31	121.1 (3)
C16—C6—H6	108.1	C33—C32—H32	119.5
C5—C6—H6	108.1	C31—C32—H32	119.5
C7—C6—H6	108.1	C32—C33—C28	119.5 (2)
N2—C7—C35	111.71 (19)	C32—C33—H33	120.2
N2—C7—C8	111.33 (19)	C28—C33—H33	120.2
C35—C7—C8	103.68 (19)	O2—C34—H34A	109.5
N2—C7—C6	106.29 (17)	O2—C34—H34B	109.5
C35—C7—C6	109.46 (19)	H34A—C34—H34B	109.5
C8—C7—C6	114.5 (2)	O2—C34—H34C	109.5
C9—C8—C7	109.2 (2)	H34A—C34—H34C	109.5
C9—C8—H8A	109.8	H34B—C34—H34C	109.5
C7—C8—H8A	109.8	O4—C35—O3	124.4 (2)
C9—C8—H8B	109.8	O4—C35—C7	121.9 (2)
C7—C8—H8B	109.8	O3—C35—C7	113.5 (2)
H8A—C8—H8B	108.3	C37A—C36A—O3	122.8 (11)
C10—C9—C14	119.5 (3)	C37A—C36A—H36A	106.6
C10—C9—C8	125.2 (3)	O3—C36A—H36A	106.6
C14—C9—C8	115.2 (2)	C37A—C36A—H36B	106.6
C11—C10—C9	119.8 (3)	O3—C36A—H36B	106.6
C11—C10—H10	120.1	H36A—C36A—H36B	106.6
C9—C10—H10	120.1	O3—C36B—C37B	100.1 (15)
C12—C11—C10	120.6 (3)	O3—C36B—H36C	111.7
C12—C11—H11	119.7	C37B—C36B—H36C	111.7
C10—C11—H11	119.7	O3—C36B—H36D	111.7
C11—C12—C13	120.2 (3)	C37B—C36B—H36D	111.7
C11—C12—H12	119.9	H36C—C36B—H36D	109.5
C13—C12—H12	119.9	C36A—C37A—H37A	109.5
C14—C13—C12	119.7 (3)	C36A—C37A—H37B	109.5
C14—C13—H13	120.2	H37A—C37A—H37B	109.5
C12—C13—H13	120.2	C36A—C37A—H37C	109.5
C13—C14—C9	120.2 (3)	H37A—C37A—H37C	109.5
C13—C14—C15	123.9 (3)	H37B—C37A—H37C	109.5
C9—C14—C15	115.8 (2)	C36B—C37B—H37D	109.5
N2—C15—C14	109.52 (18)	C36B—C37B—H37E	109.5
N2—C15—H15A	109.8	H37D—C37B—H37E	109.5
C14—C15—H15A	109.8	C36B—C37B—H37F	109.5
N2—C15—H15B	109.8	H37D—C37B—H37F	109.5
C14—C15—H15B	109.8	H37E—C37B—H37F	109.5
H15A—C15—H15B	108.2	O5—C38—H38A	109.5
C21—C16—C17	116.7 (2)	O5—C38—H38B	109.5
C21—C16—C6	124.1 (2)	H38A—C38—H38B	109.5
C17—C16—C6	119.2 (2)	O5—C38—H38C	109.5
C18—C17—C16	122.1 (2)	H38A—C38—H38C	109.5
C18—C17—H17	118.9	H38B—C38—H38C	109.5
C16—C17—H17	118.9	C3—N1—C28	130.0 (2)
C17—C18—C19	120.0 (3)	C3—N1—C1	94.42 (18)
C17—C18—H18	120.0	C28—N1—C1	129.14 (19)
C19—C18—H18	120.0	C4—N2—C7	111.06 (17)
C20—C19—O5	124.8 (3)	C4—N2—C15	111.80 (16)
C20—C19—C18	119.3 (2)	C7—N2—C15	115.71 (18)
O5—C19—C18	115.9 (3)	O6—N3—O7	124.1 (2)
C19—C20—C21	120.0 (2)	O6—N3—C5	118.8 (2)
C19—C20—H20	120.0	O7—N3—C5	117.1 (2)
C21—C20—H20	120.0	C31—O2—C34	116.7 (2)
C16—C21—C20	121.9 (3)	C35—O3—C36A	113.4 (5)
C16—C21—H21	119.1	C35—O3—C36B	119.7 (10)
C20—C21—H21	119.1	C36A—O3—C36B	17.5 (10)
C23—C22—C27	118.6 (2)	C19—O5—C38	117.4 (3)
			
N1—C1—C2—C22	119.1 (2)	C2—C22—C23—C24	−175.0 (3)
C4—C1—C2—C22	−0.6 (3)	C22—C23—C24—C25	−0.3 (5)
N1—C1—C2—C3	−1.17 (17)	C23—C24—C25—C26	−1.6 (5)
C4—C1—C2—C3	−120.9 (2)	C24—C25—C26—C27	1.6 (5)
C22—C2—C3—O1	61.1 (4)	C23—C22—C27—C26	−2.3 (4)
C1—C2—C3—O1	−177.8 (3)	C2—C22—C27—C26	174.9 (3)
C22—C2—C3—N1	−119.9 (2)	C25—C26—C27—C22	0.4 (5)
C1—C2—C3—N1	1.26 (18)	C33—C28—C29—C30	−0.6 (4)
N1—C1—C4—N2	58.1 (3)	N1—C28—C29—C30	−178.7 (3)
C2—C1—C4—N2	159.31 (18)	C28—C29—C30—C31	−0.9 (5)
N1—C1—C4—C5	179.16 (17)	C29—C30—C31—O2	−179.4 (3)
C2—C1—C4—C5	−79.7 (2)	C29—C30—C31—C32	2.2 (5)
N2—C4—C5—N3	94.8 (2)	C30—C31—C32—C33	−1.9 (5)
C1—C4—C5—N3	−31.3 (3)	O2—C31—C32—C33	179.5 (3)
N2—C4—C5—C6	−25.4 (2)	C31—C32—C33—C28	0.4 (5)
C1—C4—C5—C6	−151.54 (18)	C29—C28—C33—C32	0.9 (5)
N3—C5—C6—C16	142.32 (19)	N1—C28—C33—C32	179.0 (3)
C4—C5—C6—C16	−98.4 (2)	N2—C7—C35—O4	164.9 (2)
N3—C5—C6—C7	−91.7 (2)	C8—C7—C35—O4	−75.2 (3)
C4—C5—C6—C7	27.5 (2)	C6—C7—C35—O4	47.4 (3)
C16—C6—C7—N2	103.9 (2)	N2—C7—C35—O3	−20.1 (3)
C5—C6—C7—N2	−19.9 (2)	C8—C7—C35—O3	99.9 (3)
C16—C6—C7—C35	−135.3 (2)	C6—C7—C35—O3	−137.6 (2)
C5—C6—C7—C35	100.9 (2)	O1—C3—N1—C28	24.7 (5)
C16—C6—C7—C8	−19.4 (3)	C2—C3—N1—C28	−154.4 (2)
C5—C6—C7—C8	−143.24 (19)	O1—C3—N1—C1	177.8 (3)
N2—C7—C8—C9	49.1 (2)	C2—C3—N1—C1	−1.34 (19)
C35—C7—C8—C9	−71.1 (2)	C29—C28—N1—C3	159.1 (3)
C6—C7—C8—C9	169.71 (18)	C33—C28—N1—C3	−19.0 (4)
C7—C8—C9—C10	125.3 (2)	C29—C28—N1—C1	14.7 (4)
C7—C8—C9—C14	−51.4 (3)	C33—C28—N1—C1	−163.4 (3)
C14—C9—C10—C11	−1.5 (4)	C4—C1—N1—C3	119.2 (2)
C8—C9—C10—C11	−178.1 (3)	C2—C1—N1—C3	1.30 (19)
C9—C10—C11—C12	0.6 (4)	C4—C1—N1—C28	−87.3 (3)
C10—C11—C12—C13	0.3 (5)	C2—C1—N1—C28	154.8 (2)
C11—C12—C13—C14	−0.3 (5)	C1—C4—N2—C7	138.00 (18)
C12—C13—C14—C9	−0.5 (4)	C5—C4—N2—C7	12.9 (2)
C12—C13—C14—C15	175.9 (2)	C1—C4—N2—C15	−91.1 (2)
C10—C9—C14—C13	1.4 (4)	C5—C4—N2—C15	143.76 (19)
C8—C9—C14—C13	178.3 (2)	C35—C7—N2—C4	−114.9 (2)
C10—C9—C14—C15	−175.3 (2)	C8—C7—N2—C4	129.7 (2)
C8—C9—C14—C15	1.7 (3)	C6—C7—N2—C4	4.4 (2)
C13—C14—C15—N2	−127.0 (3)	C35—C7—N2—C15	116.2 (2)
C9—C14—C15—N2	49.5 (3)	C8—C7—N2—C15	0.8 (3)
C5—C6—C16—C21	29.0 (3)	C6—C7—N2—C15	−124.4 (2)
C7—C6—C16—C21	−90.2 (3)	C14—C15—N2—C4	−178.13 (19)
C5—C6—C16—C17	−150.5 (2)	C14—C15—N2—C7	−49.7 (3)
C7—C6—C16—C17	90.3 (3)	C6—C5—N3—O6	40.4 (3)
C21—C16—C17—C18	0.3 (4)	C4—C5—N3—O6	−76.4 (3)
C6—C16—C17—C18	179.9 (3)	C6—C5—N3—O7	−142.3 (2)
C16—C17—C18—C19	−1.4 (5)	C4—C5—N3—O7	101.0 (2)
C17—C18—C19—C20	1.2 (5)	C30—C31—O2—C34	2.4 (5)
C17—C18—C19—O5	−178.5 (3)	C32—C31—O2—C34	−179.1 (3)
O5—C19—C20—C21	179.7 (3)	O4—C35—O3—C36A	8.5 (8)
C18—C19—C20—C21	0.0 (4)	C7—C35—O3—C36A	−166.4 (7)
C17—C16—C21—C20	1.0 (4)	O4—C35—O3—C36B	−9.9 (10)
C6—C16—C21—C20	−178.6 (3)	C7—C35—O3—C36B	175.3 (9)
C19—C20—C21—C16	−1.1 (5)	C37A—C36A—O3—C35	106.6 (10)
C3—C2—C22—C23	−156.6 (3)	C37A—C36A—O3—C36B	−139 (5)
C1—C2—C22—C23	101.9 (3)	C37B—C36B—O3—C35	132.2 (15)
C3—C2—C22—C27	26.2 (3)	C37B—C36B—O3—C36A	59 (4)
C1—C2—C22—C27	−75.3 (3)	C20—C19—O5—C38	−1.1 (5)
C27—C22—C23—C24	2.3 (4)	C18—C19—O5—C38	178.5 (3)

### Hydrogen-bond geometry (Å, °)

**Table:**

*D*—H···*A*	*D*—H	H···*A*	*D*···*A*	*D*—H···*A*
C6—H6···O2^i^	0.98	2.42	3.260 (3)	143
C20—H20···O1^ii^	0.93	2.47	3.397 (3)	172
C29—H29···O3	0.93	2.59	3.442 (3)	152
C29—H29···N2	0.93	2.50	3.168 (3)	128
C33—H33···O1	0.93	2.44	3.054 (4)	123

Symmetry codes: (i) *x*, *y*+1, *z*; (ii) *x*−1/2, −*y*+1/2, −*z*.
